# TCR T cells overexpressing c-Jun have better functionality with improved tumor infiltration and persistence in hepatocellular carcinoma

**DOI:** 10.3389/fimmu.2023.1114770

**Published:** 2023-05-04

**Authors:** Mohamed S. Hussein, Qi Li, Rui Mao, Yibing Peng, Yukai He

**Affiliations:** ^1^ Georgia Cancer Center, Medical College of Georgia, Augusta University, Augusta, GA, United States; ^2^ Department of Medicine, Medical College of Georgia, Augusta University, Augusta, GA, United States

**Keywords:** TCR T cells, T cell engineering, adoptive cell therapy, hepatocellular carcinoma, tumor immunotherapy

## Abstract

**Background:**

The overall 5-year survival rate of hepatocellular carcinoma (HCC), a major form of liver cancer, is merely 20%, underscoring the need for more effective therapies. We recently identified T cell receptors (TCR) specific for the HLA-A2/alpha fetoprotein amino acids 158-166 (AFP_158_) and showed that these TCR engineered T cells could control HCC xenografts in NSG mice. However, their efficacy was limited by poor expansion, loss of function, and short persistence of the TCR T cells. Here, we studied whether overexpression of c-Jun, a transcription factor required for T cell activation, in the TCR T cells could enhance their expansion, function, and persistence in HCC tumor models.

**Methods:**

Recombinant lentiviral vectors (lv), expressing either the HLA-A2/AFP_158_-specific TCR or both the TCR and c-Jun (TCR-JUN), were constructed and used to transduce primary human T cells to generate the TCR or TCR-JUN T cells, respectively. We compared the expansion, effector function, and exhaustion status of the TCR and TCR-JUN T cells *in vitro* after HCC tumor stimulation. Additionally, we studied the persistence and antitumor effects of the TCR and TCR-JUN T cells using the HCC xenografts in NSG mice.

**Results:**

We could effectively transduce primary human T cells to express both TCR and c-Jun. Compared to the HLA-A2/AFP_158_ TCR T cells, the TCR-JUN T cells have better expansion potential in culture, with enhanced functional capacity against HCC tumor cells. In addition, the TCR-JUN T cells were less apoptotic and more resistant to exhaustion after HepG2 tumor stimulation. In the HCC xenograft tumor model, c-Jun overexpression enhanced the TCR T cell expansion and increased the overall survival rate of the treated mice. Importantly, the TCR-JUN T cells were less exhausted in the tumor lesions and demonstrated enhanced tumor infiltration, functionality, and persistence.

**Conclusion:**

c-Jun overexpression can enhance the expansion, function, and persistence of the A2/AFP_158_ TCR engineered T cells. The c-Jun gene co-delivery has the potential to enhance the antitumor efficacy of AFP specific TCR T cells when treating patients with HCC.

## Background

Hepatocellular carcinoma (HCC) accounts for 90% of primary liver cancer (1), the sixth most common cancer in the world (2). In the United States, the liver cancer incidence rate doubled from 1999 to 2016 (3). Liver cancer is the third-leading cause of cancer death, highlighting the need for novel therapies. Today, immunotherapy has become a frontline cancer treatment (4–6). Its efficacy relies on tumor-infiltrating T cells, which unfortunately do not exist in most solid tumors (7). Engineering a patient’s T cells with tumor-specific receptors, such as T cell receptors (TCRs) (8, 9) and chimeric antigen receptors (CARs) (10–12), can increase their T cell’s tumor targeting capability, providing the much-needed tumor-specific T cells to achieve antitumor effects. TCR enables T cells to bind the tumor antigen presented on MHC molecules. Thus, TCR T cells can recognize both surface and intracellular antigens. Since more than 90% of tumor antigens are derived from intracellular protein, TCR T cells can target a broader antigen repertoire (13).

Recently, we identified several novel TCRs specific to the HLA-A2 restricted Alpha-fetoprotein epitope (AFP_158_) and showed that human T cells engineered with such TCR genes could recognize and kill AFP^+^ HepG2 tumor cells, which resulted in regression of HCC xenografts in immunocompromised NSG mice (9). In addition, because the HLA-A2/AFP_158_ TCR did not show cross reactivity with off-target antigens (14), one of the TCRs has been selected (15) to enter into clinical trials (NCT03971747). However, the persistence of TCR T cells is short-lived, and the TCR T cell expansion *in vivo* is very limited, if at all. The inability of TCR T cells to undergo *in vivo* expansion suggests that either the tumor cells are insufficient to engage TCR T cells and initiate T cell expansion or the TCR T cells are exhausted and in a terminally differentiated stage and have lost their proliferation potential due to chronic stimulation in the tumor microenvironment (TME) (16). Different approaches are being studied to improve the outcome of adoptive cell therapy (ACT). These include increasing TCR affinity to target cells (17, 18), armoring TCR T cells with pro-inflammatory cytokines such as IL-12 or IL-18 (19–22), and enhancing TCR signaling with the addition of the CD28 or 4-1BB intracellular domains (ICDs) in the CD3 ζ chain (23, 24). Additionally, overexpression of transcription factor was explored to increase T cell’s activation and function (25).

c-Jun is a member of the AP-1 family of transcription factors, which is required for T cell activation, and its activity is strongly augmented by TCR signaling through Jun kinase (JNK) (26, 27). During T cell activation, c-Jun forms a dimer with another AP-1 family member: c-Fos. The c-Jun/c-Fos heterodimer binds to the AP-1 binding site of the promoter of several target genes required for T cell activation and proliferation, like IL-2 and cell cycle genes, to drive their transcription. In addition, they form ternary complexes with NFAT; the NFAT/c-Jun/c-Fos heterotrimer drives the transcription of IL-2 and other effector genes (28–30). However, the different AP-1 family members may be out of balance in the exhausted T cells, with more JUNB, BATF3, and IRF4, and less c-Jun. A recent study showed that the exhausted CAR-Ts were characterized by depletion of the canonical AP-1 c-Jun/c-Fos dimer. Importantly, engineering CAR-Ts to overexpress c-Jun enabled them to resist exhaustion, thus improving the antitumor potency of CAR-Ts (25). Since lack of T cell activation and loss of effector function represent hurdles for successful TCR-T cell therapy (31), we studied whether overexpression of c-Jun would generate better expansion, improve effector functions, and increase persistence of the AFP_158_ specific TCR T cells in treating HCC xenografts. Our data showed that c-Jun overexpression enhanced the functionality and persistence of TCR T cells, with more resistance to exhaustion and activation-induced cell death. More TCR-JUN T cells were also found in the tumor lesions. The TCR-JUN T cells had better function compared to TCR T cells, which extended mouse survival. Thus, engineering T cells to overexpress c-Jun could be a potential approach to enhance TCR T cell cancer therapy.

## Materials and methods

### Cells

The Jurkat, HEK293T, and HepG2 cells were purchased from American Type Culture Collection (ATCC) and cultured according to recommendation. No more than 10 passages of the cells were used to assure authenticity. HepG2-Luc and HepG2-GFP cells were created in lab, as described (9).

### Mice and tumor models

The NOD-*scid* IL2Rgamma^null^ (NSG) mice were purchased from Jackson Laboratory. Animal protocol is approved by IACUC of Augusta University. 8-10-week-old mice were inoculated subcutaneously (SC) with the indicated number of HepG2-Luc. Tumors were measured by bioluminescent intensity (BLI) using the Ami X spectral imaging system from Spectral Instruments. BLI values (total photons/s) were analyzed using AmiView software.

### Construction of lentiviral vectors (lv) encoding TCRs

The methods for A2/AFP_158_-specific TCR construction were previously described (9). The C region of the TCRα chain and the C2 region of the TCRβ chain were used to create the full-length TCRs. A P2A (porcine teschovirus-1 2A) sequence (32) was inserted between the TCR α and β chains. For TCR-JUN, A T2A (thosea asigna virus 2A) sequence (33) and c-Jun gene were inserted behind the TCRβ chain. Finally, the entire expression cassette was inserted behind the EF1α promoter in pCDH lv (Addgene). Lvs were produced as previously described (34, 35), and the titers were determined by transducing Jurkat cells.

### T cell isolation and transduction

Healthy human peripheral blood mononuclear cell (PBMC) buffy coats were purchased from Shepeard Community Blood Center in Augusta, GA, and separated by Ficoll-Paque PREMIUM (GE Healthcare, Cat#: 17-5442-02). Primary human T cells were isolated using the EasySep Human T Cell Isolation Kit (StemCell Technologies, Cat#: 17951) according to the manufacturer’s protocol. Human T cells were activated with Dynabeads™ Human T-Activator CD3/CD28 (Gibco, Thermo Fisher Scientific, Cat#: 11132D) using 1.5:1 beads to cell ratio for 24 hours and were transduced by lvs at 30 Multiplicity of Infections (MOI) as previously described (8). For transduction of T cells, lvs was calculated according to the MOI and total number of cells. For each 1 mL of lvs, 16 µg of Polybrene Transfection Reagent (MilliporeSigma, Cat#: TR-1003-G) was added to the T cell culture; the mock non-transduced T cells were only cultured with the same concentration of polybrene without lvs. The cells were centrifuged at 2000 x g for 2 hours at 32°C and incubated at 37°C. After 8-10 hours, the cells were diluted with RPMI-1640 complete medium (supplemented with 10% fetal bovine serum, 10 mM HEPES, 1x MEM amino acids, 1x 2-mercaptoethanol, 1% Antibiotic-Antimycotic) with 40 UI/ml of IL-2 (PeproTech, Cat#: 200-02) to bring the polybrene concentration to 4 µg/mL. The beads were removed by magnet after another 24 hours. The cells were maintained at 0.5 × 10^6^–1 × 10^6^ cells per mL in RPMI-1640 complete medium with 40 UI/ml of IL-2.

### ELISA and lactate dehydrogenase (LDH) assay

HepG2 cells (1 × 10^4^/well) were grown on a 96-well flat bottom plate in triplicate overnight, and TCR T cells were then added at indicated effector/target (E/T) ratios. The supernatants were collected after 20 hours and were analyzed for IL-2 and IFNγ by ELISA (BioLegend) and for cytotoxicity by LDH activity (Promega).

### IncuCyte live-cell analysis

The cytotoxicity of TCR T cells was also analyzed by IncuCyte real-time quantitative live-cell imaging and analysis (Sartorius). HepG2-GFP^+^ cells (1×10^4^/well) were plated in triplicate on a 96-well flat bottom plate and grown overnight. TCR T cells were added at the indicated E/T ratio. Pictures were taken every 3 hours using the IncuCyte SX1 Live-Cell analysis system for 3 days. 5 images per well were collected at each time point. The total GFP area was assessed at each time point by IncuCyte analysis software.

### Tumor antigen-stimulation assay

HepG2 cells (1×10^6^/well) were seeded into 6-well plate and grown overnight. After 24 hours, the medium was removed, and TCR T cells (day 7 after lv transduction) were added at 0.5:1 E/T ratio and co-cultured for up to 6 days in RPMI-1640 complete medium with and without 40 UI/mL of IL-2. Duplicate wells were plated for each condition. Mock non-transduced T cells were included as controls. TCR T cells’ expansion was calculated, and the expression of exhaustion markers and apoptosis of TCR T cells was assessed by flow cytometry. Cells were counted with Countess II™ Automated Cell Counter using trypan blue stain (0.4%) (Invitrogen, Cat#: T10282) in Countess™ Cell Counting Chamber Slides (Invitrogen, Cat#: C10228).

### Flow cytometry

Human T cells were stained with the indicated antibodies and analyzed on flow cytometers of LSR (BD Bioscience) or NovoCyte Quanteon (Agilent). For some staining, Zombie Green Fixable Viability Kit (BioLegend) was added to exclude dead cells. The antibodies included BioLegend CD45, CD8, CD4, CD3, TCRb, PD1, LAG-3, TIM-3, Annexin-V, Bcl-2, IL-2, and IFNγ. The c-Jun (60A8), rabbit (DA1E) mAb IgG XP isotype control, and anti-rabbit IgG (H+L), F(ab’)2 fragment were from Cell Signaling. Unless otherwise indicated, all antibodies were used at 1:200 dilution in PBS. The c-Jun (60A8) and rabbit (DA1E) mAb IgG XP isotype control were diluted in antibody dilution buffer (0.5% BSA in 1x PBS) and used according to manufacturer’s recommendation (Cell Signaling).

### Adoptive cell transfer (ACT)

TCR or TCR-JUN T cells (10-12 days after transduction) of the indicated numbers were injected into tumor-bearing mice intravenously (IV). Human T cells and TCR^+^ T cells were monitored in the blood by staining with indicated antibodies anti-human CD45 and anti-mouse TCRbeta (the TCR specific for HLA-A2/AFP_158_ were identified from immunized mice (9). Mouse CD45 cells were stained as an internal reference.

### Tumor infiltrating lymphocytes (TILs)

For *ex vivo* analysis of tumor infiltrating TCR T cells, tumor tissue was collected, washed in PBS, and mechanically dissociated and digested in RPMI media supplemented with 0.1% collagenase type V (Sigma C9263), 0.1% hyaluronidase (Sigma H6254), and 100 unit/mL DNase I (Sigma D4527)) for 30-45 minutes at 37°C. After RBCs lysis, the single cell suspension was ready for analysis by flow cytometry and for cytotoxicity by IncuCyte Live-Cell analysis system.

### Intracellular cytokine staining

To study the cytokine production, TIL TCR T cells were prepared and co-cultured with 2.5×10^5^ HepG2 tumor cells for 20 hours in a 24-well plate according to the percentage of TCR^+^ T cells and the desired E/T ratios. Monensin (BioLegend) was added to the co-culture for the last 4 hours. Intracellular cytokine staining of IL2 and IFNγ was performed according to the manufacturer’s instruction (BioLegend).

### IHC staining

Tumor tissues were collected and fixed in 10% Buffered Formalin Phosphate (Fisher Chemical, Cat#: SF100-4) for 24 hours and then replaced with 70% ethanol. The paraffin-embedded tissues were sectioned and stained by anti-CD8 antibody (Sino Biologicals, Cat#: 10980-T24) and HRP conjugated secondary anti-Rabbit IgG, which was revealed by TMB substrate.

### Bulk RNA sequencing

The TCR T cells after *in vitro* stimulation with HepG2 tumor cells were isolated by cell sorting based on the hCD45 and TCRb markers. Total RNA was isolated by Quick-RNA Microprep Kit (Zymo Research) and then mRNA was isolated by oligo-dT. The RNA libraries were prepared from the purified mRNA and sequenced by Novogene. The paired-end 150bp raw sequencing reads were examined by FastQC v0.11.8. There were minimal 20 million sequence reads for each sample. Adaptor sequences and low-quality bases were trimmed using Trim Galore! v0.6.3. The cleaned reads were mapped to reference genome (mm10) using STAR aligner v2.7.8a. The QC and mapping steps were performed in Galaxy server (use.galaxy.org). The sequence counts for each gene were collected by Rsubread featureCounts functions v1.22.2. The differential gene expression analysis was performed using DESeq2 v1.32.0. The heatmaps were generated using ComplexHeatmap v2.8.0 in R v4.1.

### Statistical analysis

Statistical analyses were performed using student two-tailed *t*-test or 2-way ANOVA in the Prism software (GraphPad Inc.). Survival curves were compared using the log-rank Mantel–Cox test. Data points represent biological replicates and are shown as the mean ± standard error of mean (SEM) or mean ± standard deviation (SD) as indicated in the figure legends. Significance was assumed with *, P < 0.05; **, P < 0.01; ***, P < 0.001; and ****, P < 0.0001.

## Results

### c-Jun overexpression enhances the expansion of T cells

**Figure 1:**
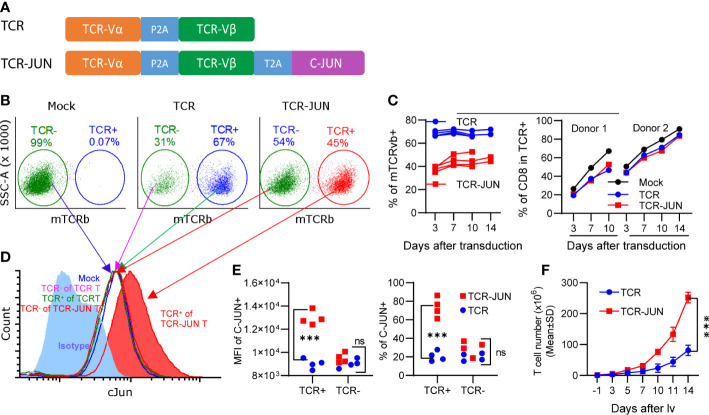
Overexpression of c-Jun increases expansion of human T cells. **(A)** showed the recombinant lv co-expressing the AFP158-specific TCR and c-Jun. **(B)** Representative transduction of human T cells with recombinant lvs was presented. **(C)** presented the kinetics of TCR and TCR-JUN transduction (4 donor T cells) and the change of CD8 percentage (2 donor T cells) among the TCR or TCR-JUN positive T cells. For Mock group, the % of CD8 among the total T cells was shown. **(D, E)** Increased level of c-Jun was observed in the TCR+ T cells after transduction with the TCR-JUN lv. The isotype staining was used to set up the gate for measuring the % of c-Jun. The entire population of TCR- or TCR+ T cells were gated and the MFI of c-Jun was measured and presented. **(F)** The kinetics of T cell expansion after transduction of TCR or TCR-JUN was shown. Data were from four donor T cells. Each point represents the mean ± SEM. ***p <0.001. ns, not significant.

In the first experiment, we studied the effects of c-Jun overexpression on human T cell expansion *in vitro*. To this end, we first constructed 2 lentiviral vectors (lvs) that expressed the TCR specific for HLA-A2/AFP_158_ and the c-Jun separately (**Supplemental Figure S1A**). Human T cells were isolated by negative selection to a purity of >97% (**Supplemental Figure S1B**). However, only a small fraction of T cells could be co-transduced by both TCR-lv and c-Jun-lv (**Supplemental Figures S1C, D**). To improve the transduction of T cells with both TCR and c-Jun simultaneously, we generated the lv that co-expresses AFP_158_ TCR and c-Jun (TCR-JUN) (**Figure 1A**). This TCR and c-Jun overexpression in one vector enabled all TCR^+^ T cells to express the c-Jun at the same time. We found that the transduction efficiencies of TCR and TCR-Jun T cells were approximately 67% and 45%, respectively (**Figure 1B**). The percentage of TCR^+^ T cells remained steady over time (**Figure 1C**). Compared to the mock non-transduced T cells, transduction of T cells with TCR or TCR-JUN had little impact on the ratio of CD8 T cells (**Figure 1C**). Intracellular staining showed that human T cells expressed a basal level of c-Jun, which did not change after exogenous TCR transduction (**Figure 1D**). After transduction, the TCR^+^ T cells had the same basal level of c-Jun as the TCR^-^ (negative) T cells after either TCR-JUN-lv or TCR-lv transduction; this level was also similar to that of the mock non-transduced T cells. In contrast, the c-Jun level in TCR^+^ T cells after TCR-JUN lv transduction was 3-folds higher than that in the TCR negative T cells (**Figures 1D, E**). Importantly, the number of total T cells in the TCR-JUN-lv transduced group was significantly higher than that of the TCR-lv group, suggesting that the c-Jun overexpression in the TCR transduced T cells enhanced the expansion of total human T cells (**Figure 1F** and **Supplemental Figure S2**). Together, this data shows that the recombinant lv co-expressing TCR and c-Jun can efficiently transduce human T cells and that overexpression of c-Jun also enhanced TCR T cell expansion in culture.

### c-Jun overexpression does not affect the cytotoxicity but significantly increases the cytokine production of TCR T cells

**Figure 2:**
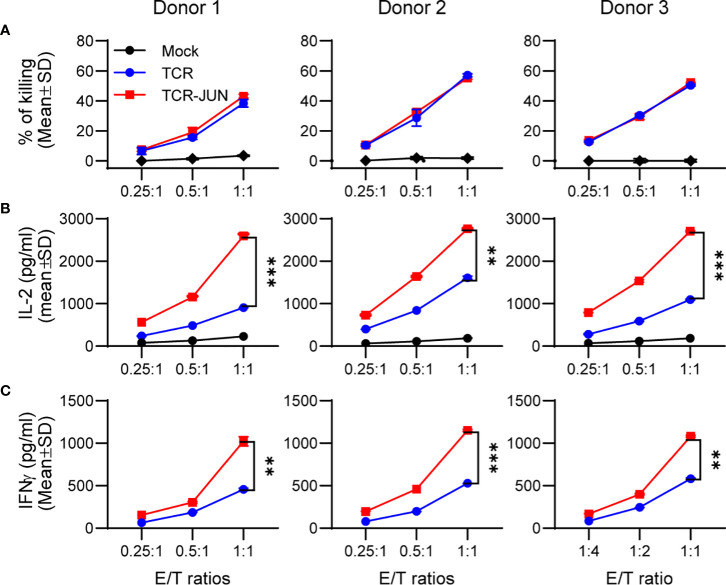
Overexpression of c-Jun significantly increases IL2 and IFNγ of TCR T cells without affecting cytotoxicity. Donor T cells were transduced with either TCR or TCR-JUN genes. The TCR or TCR-JUN T cells were co-cultured with HepG2 tumor cells at indicated E:T ratios for 20hrs. The media were collected and measured for cytotoxicity by LDH assay **(A)**, and IL2 **(B)** and IFNγ **(C)** by ELISA. Each point represents the mean ± SD of 3 repeating wells. Statistical analysis was done by two-way ANOVA. **p<0.01; ***p<0.001.

We next investigated whether c-Jun overexpression would affect the effector function (cytotoxicity and cytokine production) of TCR T cells. To this end, the TCR and TCR-JUN T cells were co-cultured with HepG2 tumor cells at different effector/target (E/T) ratios for 20 hours. Using LDH assay, we found that the TCR-JUN T cells maintained similar cytotoxicity against HepG2 tumor cells as the TCR T cells (**Figure 2A**). The cytotoxicity of TCR and TCR-JUN T cells was additionally studied by using the IncuCyte Live-cell analysis, which monitored the growth kinetics of target tumor cells. We found that the TCR-JUN T cells had similar, if not higher than, cytotoxicity as TCR T cells (**Supplemental Figure S3**). However, the TCR-JUN T cells showed 2-3 folds increase in IL-2 and IFNγ production compared to the TCR T cells (**Figures 2B, C**). These results indicate that c-Jun overexpression may not significantly alter the cytolytic capacity of TCR T cells but can increase the production of IL2 and IFNγ cytokines.

### c-Jun overexpression enhances the expansion and reduces the exhaustion and apoptosis of the TCR T cells driven by target tumor cells

**Figure 3:**
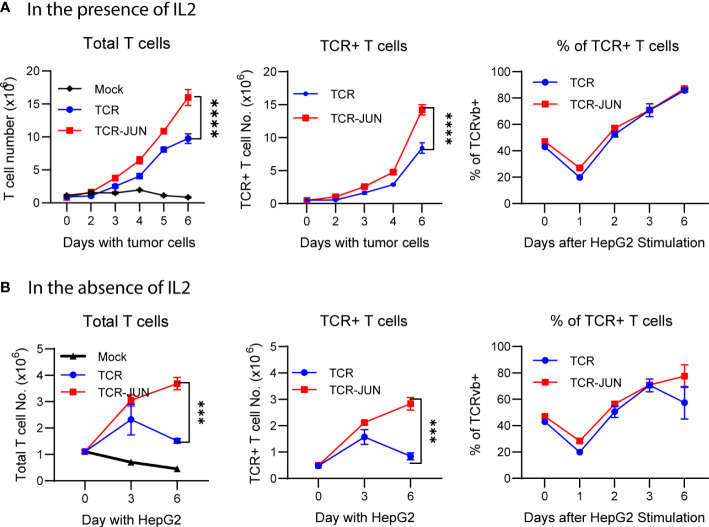
c-Jun enhances the TCR T expansion driven by target tumor cells. The number of total T cells, TCR+ T cells, and the % of TCR+ T cells at different time points after HepG2 tumor cells stimulation in the presence of IL2 **(A)** or in the absence of IL2 **(B)** were presented. Each point represents the mean ± SEM of 4 repeating wells from 2 different donor T cells. ****p <0.0001, ***p <0.001.

The antitumor effect of TCR T cells is also related to their exhaustion status and persistence, in addition to their effector function. Thus, we studied the effect of c-Jun overexpression on the expansion, survival, and exhaustion of AFP_158_ TCR T cells driven by tumor antigen stimulation. The TCR-JUN and TCR T cells were co-cultured with HepG2 tumor cells, and the number of TCR T cells was counted at different time points. We found that, compared to TCR T cells, the TCR-JUN T cells showed significant increase in the number of total T cells and the TCR+ T cells even though the % of TCR^+^ T cells are similar on day 6 after stimulation with tumor cells (**Figures 3A, B**). In the absence of exogenous IL2 in the media, the effect of c-Jun expression on the expansion of TCR T cells was even more significant (**Figure 3B**). In fact, in the absence of exogenous IL2 in the culture medium, the number of total T cells and the TCR^+^ T cells began decreasing after day 3. In contrast, the TCR-JUN T cells continued to expand, even on day 6 after stimulation with tumor cells.

We next used Annexin V staining to determine the apoptotic status of TCR T cells after tumor cell stimulation. Compared to the TCR T cells, the TCR-JUN T cells showed a significantly lower Annexin-V staining, almost 1.5-folds (**Supplemental Figure S4**). The data from 4 different donor T cells showed that TCR-JUN T cells are less apoptotic than TCR T cells after tumor antigen stimulation. Then, we examined the exhaustion markers, programmed cell death protein 1 (PD-1), lymphocyte activation gene 3 (LAG-3), and T cell immunoglobin and mucin-domain containing-3 (TIM-3) on the surface of TCR-JUN and TCR T cells following tumor antigen stimulation (**Supplemental Figure S5**). While no difference of PD1 level was observed, the TCR-JUN T cells showed significantly lower expression of LAG3 and TIM3 compared to the TCR T cells.

In addition, we studied the effect of c-Jun expression on TCR T cells by bulk RNA-seq analysis. The TCR and TCR-JUN T cells were sorted after *in vitro* stimulation with HepG2 tumor cells, and the RNA-seq analysis was conducted with 2 donor T cells. The RNA-seq data is in line with the immunological data by showing that overexpressing c-Jun decreased the expression of co-inhibitory receptors and exhaustion-associated transcription factors, such as LAG3, NR4A and EOMES, in the TCR-JUN T cells (**Supplemental Figure S6**) (36). The KLRC1 (Killer Cell Lectin Like Receptor C1), which encodes the inhibitory receptor NKG2A (natural killer cell lectin-like receptor isoform A), was upregulated in the TCR T cells. NKG2A was found to be expressed by exhausted CD8^+^ T cells, and its blockage restored the effector function of tumor-specific exhausted CD8^+^ T cells (37). In contrast, we noticed upregulation of GSDMD in the TCR-JUN T cells, which is important to initiate an inflammatory cascade through the release of the pro-inflammatory cytokines IL-1ß and IL-18 (38). RTKN2 protein is overexpressed in the TCR-JUN T cells, which is known to confer resistance to apoptosis in T-cells by regulating pro-apoptotic and anti-apoptotic BCL-2 genes (39). Although the conclusion is less persuasive with data from only 2 donor T cells, the RNA-seq data further validate c-Jun’s effect on TCR T cells.

In summary, the immunological staining data and RNAseq analysis demonstrate that the TCR-JUN T cells have significantly higher tumor driven expansion with less exhaustion and more resistance to activation-induced cell death than TCR T cells *in vitro*.

### c-Jun overexpression enhances TCR T cell expansion *in vivo* and improves the overall survival of the treated mice

**Figure 4:**
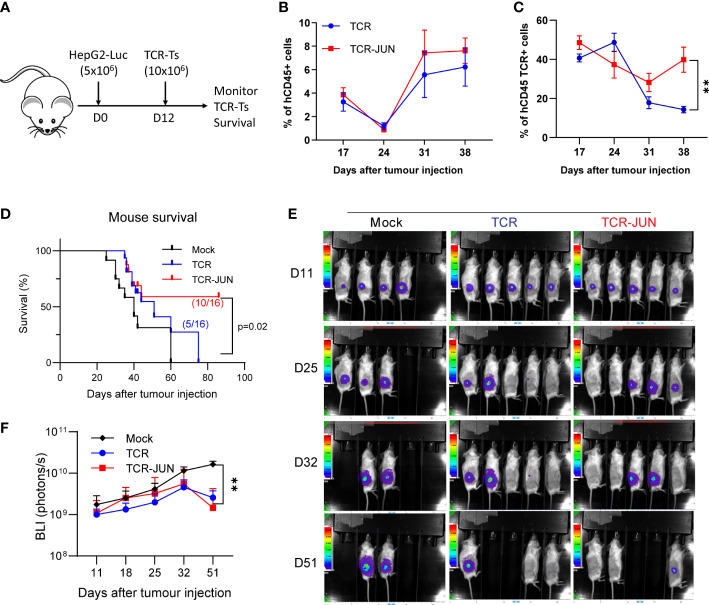
c-Jun enhances the expansion of TCR+ cells and survival of HCC xenograft bearing mice. **(A)** NSG mice were injected subcutaneously with 5 × 10^6^ HepG2-Luc tumor cells, and then 10 × 10^6^ Mock, TCR or TCR-JUN TCR+ T cells were given intravenously on day 12. **(B, C)** The % of hCD45+ and TCR+ T cells in the blood of tumor bearing mice after ACT. Each point represents the mean ± SEM. **p <0.01. **(D)** Kaplan Meier survival curve of Mock (n=12), TCR (n=16), and TCR-JUN (n=16) treated mice. **(E, F)** The kinetics and BLI of the SC tumors of NSG mice after ACT. Each point represents the mean ± SEM. **p <0.01.

In this experiment, we utilized the subcutaneous (SC) HCC xenograft tumor model in NSG mice to study whether the c-Jun overexpression would enhance the *in vivo* expansion and persistence of TCR T cells. Five million HepG2-Luc cells were inoculated subcutaneously to establish SC tumors for 12 days before treatment with TCR T cells or TCR-JUN T cells **(** **Figure 4A**). We found that, compared to the TCR T cell treated mice, the % of TCR^+^ T cells were significantly higher (2-folds more) in the peripheral blood of TCR-JUN T cells treated mice on day 38 after tumor injection (26 days after ACT), even though the % of total human T cells was only slightly higher (**Figures 4B, C**). Additionally, the overall survival time of the mice treated with TCR-JUN T cells was significantly longer than the mice treated with TCR or mock non-transduced T cells (**Figure 4D**). The data suggests that TCR T cells engineered to overexpress c-Jun have better expansion and persistence *in vivo*, resulting in enhanced mouse survival. However, we did not observe a significant difference in the anti-tumor effect between TCR T cells and TCR-JUN T cells (**Figures 4E, F**) even though the tumor BLI of the TCR-JUN T cell treated group was slightly lower.

### TCR-JUN T cells have better infiltration and persistence in the tumor mass compared to TCR T cells

**Figure 5:**
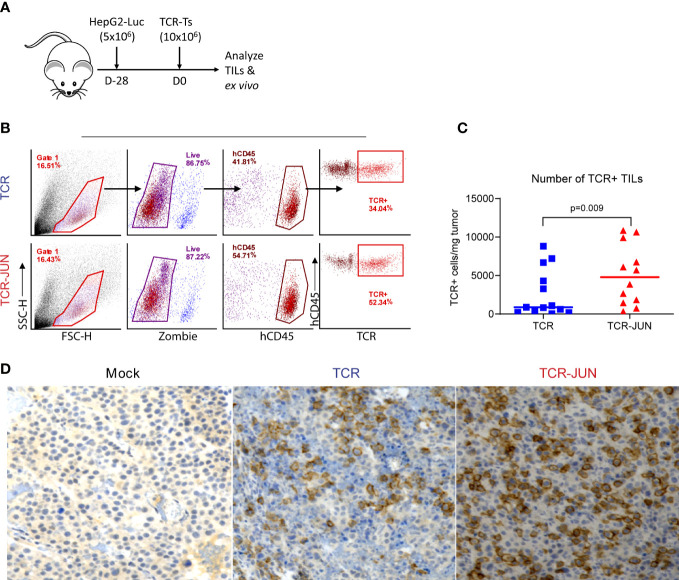
TCR-JUN T cells have more infiltration into tumor mass compared to TCR T cells. **(A)** NSG mice were injected subcutaneously with 5 × 10^6^ HepG2-Luc tumor cells, and then 10 × 10^6^ Mock, TCR or TCR-JUN TCR+ T cells were given intravenously on day 28. **(B, C)** The gating strategy for enumerating the tumor infiltrating TCR+ T cells **(B)** and the % of hCD45+ TCR+ T cells **(C)**. The viable cells from the single tumor cell suspension were counted by Trypan blue and further determined by Zombie staining. The TCR+T cell number in the tumor was calculated by the total viable cells in the tumor x % of hCD45 x % of TCR+/mg of tumor. **(D)** Representative IHC staining of tumor infiltrating CD8 T cells at Day 13 after ACT using 10x magnification. The data showed that more TCR+T cells were found in the TCR-JUN treated tumors.

We next examined the tumor infiltrating TCR and TCR-JUN T cells to test the hypothesis that c-Jun overexpression would increase their persistence in solid tumor mass. To obtain sufficient tumor infiltrating TCR T cells, we developed large SC tumors (28 days, 1 centimeter in diameter) before ACT (**Figure 5A**). The single cell suspension from the tumor lesions was analyzed by flow cytometry, and it revealed significantly more hCD45 TCR^+^ T cells in the tumors treated with TCR-JUN T cells compared to TCR T cells. On day 13 after ACT, we found that there were significantly more TCR+ T cells in the tumor treated with TCR-JUN T cells, almost 5-folds, than in the TCR T cell-treated tumors (**Figures 5B, C**). Moreover, immunohistochemistry staining demonstrated far more tumor infiltrating human CD8 T cells in the tumor sections treated with TCR-JUN T cells (**Figure 5D**). These findings indicate that TCR-JUN T cells accumulate and persist longer at the tumor site than TCR T cells after ACT.

### TCR-JUN T cells in the tumors are less exhausted and more functional than TCR T cells

**Figure 6:**
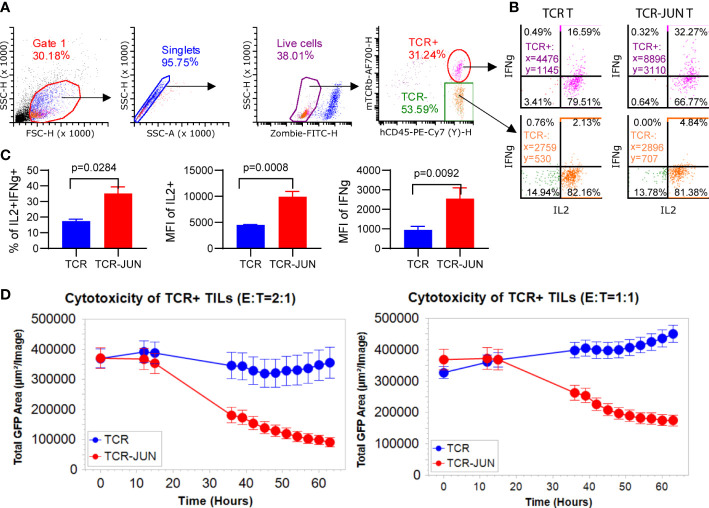
TCR-JUN T cells are more functional inside solid tumor lesions. **(A)** The gating strategy of the single cell suspensions from treated tumors. **(B, C)** ICS of the IL2 and IFNγ in the tumor infiltrating TCR+ T cells from the TCR-T and TCR-JUN T treated tumors (5 tumors in each group). Data represent mean ± SEM. **(D)** Killing assay of tumor infiltrating TCR+ T cells isolated 13 days after treatment with TCR or TCR-JUN T cells. Each point represents the mean ± SD of three repeating wells (15 pictures).

Since the *in vitro* data showed that TCR-JUN T cells were less exhausted and more resistant to tumor induced cell death than TCR T cells, we wanted to test the hypothesis that TCR-JUN T cells would be more resistant to exhaustion and would maintain better effector function in solid tumor lesions. First, we found that the tumor infiltrating TCR-JUN T cells are less exhausted than the TCR T cells, with significantly less expression of double positive PD-1 and LAG-3 inhibitory receptors on the surface of TCR-JUN T cells (**Supplemental Figure S7A**). In addition, the tumor infiltrating TCR-JUN T cells showed higher expression of anti-apoptotic Bcl-2 (**Supplemental Figure S7B**), indicating that TCR-JUN T cells are less apoptotic than TCR T cells. Next, we tested the effector function of tumor infiltrating TCR and TCR-JUN T cells by assessing their cytokine production and cytotoxicity. The tumor infiltrating T cells in the single cell suspension from treated tumors were co-cultured with HepG2 tumor cells to measure their cytokine production and cytotoxicity. In agreement with the less exhaustion and apoptosis, tumor infiltrating TCR-JUN T cells produced nearly 2-folds of IL-2 and IFNγ (**Figures 6A–C**). In addition, the tumor-infiltrating TCR-JUN T cells maintained their cytotoxicity against HepG2 tumor cells and showed significantly enhanced cytotoxicity compared to TCR T cells (**Figure 6D**). In conclusion, our data shows that, compared to TCR T cells, more TCR-JUN T cells are found in the solid tumor mass, they are less exhausted and apoptotic, and maintain better effector function.

## Discussion

Adoptive transfer of TCR T cells has demonstrated great potential in treating solid tumors in clinical trials (40). However, like all ACT, its efficacy is limited by the short persistence and the exhaustion of the transferred T cells (17, 25, 31, 41, 42). In this study, we investigated whether overexpression of the transcription factor c-Jun would enhance the function and persistence of TCR T cells against HCC. We were able to co-express c-Jun in the TCR T cells specific for the HLA-A2/AFP_158_ complex (TCR-JUN T cells). We found that c-Jun overexpression enhanced T cell expansion driven by anti-CD3/CD28 magnetic beads, as well as TCR T cell expansion by tumor antigenic stimulation. The TCR-JUN T cells produced higher amounts of IL-2 and IFNγ, without significantly altering the cytotoxicity. Importantly, after ACT into tumor bearing mice, more TCR-JUN T cells accumulated in the tumor lesions, which were functional and associated with better overall survival of the treated mice. These findings are in agreement with previous reports that c-Jun overexpression provides CAR-T cells resistance to exhaustion (25).

c-Jun is important for the transcription of several effector genes required for T cell activation and functions, such as IL-2 (30). The c-Jun overexpression could enhance T cell activation by displacing inhibitory complexes of other AP-1 and IRF family members that drive T cells towards exhaustion and terminal effector differentiation (25, 43, 44). Consistent with these previous findings, the AFP TCR T cells with c-Jun overexpression from the tumor mass were less exhausted and less apoptotic and were better able to maintain their function of killing target tumor cells and producing more cytokines of IL2 and IFNγ. The cytotoxicity of tumor infiltrating T cells was done with freshly prepared single cell suspension of the tumor lesions because we could not obtain sufficient TCR T cells to compare to TCR-JUN T cells after overnight co-culture, further suggesting that the TCR T cells in the absence of c-Jun are more prone to activation induced cell death.

The c-Jun overexpression may not significantly affect T cell growth, as the percentage of TCR-JUN T cells did not change with time of culture (**Figure 1C**). Instead, the enhanced expansion of the TCR-JUN T cells may be due to the multiple indirect effects of c-Jun expression. One significant effect of c-Jun overexpression in the TCR T cells is the increased production of IL2, which can support T cell growth. The decreased exhaustion and apoptosis of TCR-JUN T cells may also contribute to their higher expansion and persistence. This may also explain the higher cytotoxicity observed in the IncuCyte real time cell analysis assay. Different from LDH assay, which last only 20 hours, the IncuCyte real time cell analysis lasts 3 days, which provides sufficient time for TCR-JUN T cells to grow in the presence of self-produced IL2. Starting with the same number of TCR+ T cells, the overexpression of c-Jun would allow more TCR+ T cells to accumulate due to the higher IL2 expression. Thus, the overall effects of TCR-JUN T cell on target tumor cells are stronger than that of the TCR T cells.

The persistence of T cells usually correlates with the efficacy of the ACT (40, 45, 46). The TCR-JUN T cells showed more infiltration into solid tumor masses and enhanced expansion *in vivo.* This may account for the better overall survival rate of tumor-bearing mice. However, a surprising finding in our study was that we did not observe significant differences in terms of antitumor effects between ACT of the TCR and TCR-JUN T cells. This may relate to the suppressive tumor microenvironment (TME), which may play an important role in suppressing the tumor infiltrating T cells and limiting their ability to exert their effector function. Thus, in addition to the provision of tumor specific T cells, creating a favorable TME is needed for TCR T cells to exert their function to achieve antitumor effects. Future work using immune-competent mice will help to study if c-Jun overexpression would enhance the ACT in the immunosuppressive TME in solid tumors.

During T cell exhaustion, continuous TCR signaling from persistent antigen exposure results in the induction of IRF4, BATF, and NFAT transcription factors, with continuous upregulation of co-inhibitory receptors such as PD-1, LAG-3, and TIM-3. The co-inhibitory signals disrupt c-Jun activation, resulting in diminished expression of effector cytokines, whereas NFAT activation remains undisrupted to form ternary complexes with IRF4 and BATF at the enhancer and promoters of exhaustion-associated genes (25, 30). CAR-Ts engineered to overexpress c-Jun were able to overcome exhaustion by displacing JUNB/BATF3 and IRF4 complexes at the promoters of exhaustion-associated genes (25). Our group has also showed that low-avidity CAR-Ts can resist exhaustion and have better tumor infiltration (10). Thus, c-Jun overexpression could enhance T cells infiltration capacity by rendering them exhaustion-resistant.

c-Jun has been described as an oncogene linked to a range of cancers (47, 48). Before taking the c-Jun expressing TCR T cells into clinical trials, the safety of such TCR T cells needs to be studied. In our study, we did not observe any evidence of transformation. However, this may relate to the short duration of the studies. Tumor-free mice would develop graft-versus-host diseases beyond 2 months after ACT. Thus, further studies will be needed to exclude any toxicities, especially the tumorigenesis that may arise from c-Jun overexpression in the T cells. From this perspective, the overexpression of suicide markers such as truncated EGFR (huEGFRt) (49) or RQR8 (50) on TCR-JUN T cells will allow selective elimination of the administered T cells by antibody Cetuximab or Rituximab respectively, in case of toxicity.

In conclusion, our study demonstrated that the c-Jun overexpression improves the expansion and persistence of TCR-Ts *in vitro* and *in vivo*, with more resistance to exhaustion and apoptosis and better effector function. Taken together, c-Jun can potentially enhance the efficacy of TCR T cells.

## Data availability statement

The TCR sequence is included in the Patent title “Human alpha fetoprotein-specific murine T cell receptors and uses thereof”. The Patent Number 11041011. The c-Jun sequence is from Uniprot P05412 (https://www.uniprot.org/uniprotkb/P05412/entry). Readers can also contact corresponding authors to acquire these 2 sequences.

## Ethics statement

The animal study was reviewed and approved by IACUC of Augusta University.

## Author contributions

YH and MH formulated the hypothesis and designed and conducted most of the *in vitro* and *in vivo* experiments. QL, RM, and YP were involved in constructing and producing lentiviral vectors, animal care, and animal studies. MH and YH wrote the manuscript. QL, RM, and YP all read and agree with the manuscript. All authors contributed to the article and approved the submitted version.
