# Supplementary figures and images for: TCR T cells overexpressing c-Jun have better functionality with improved tumor infiltration and persistence in hepatocellular carcinoma
Source: Front Immunol. 2023 May 4;14:1114770. doi: 10.3389/fimmu.2023.1114770 (PMC10192869; doi:10.3389/fimmu.2023.1114770)

## Slide 1
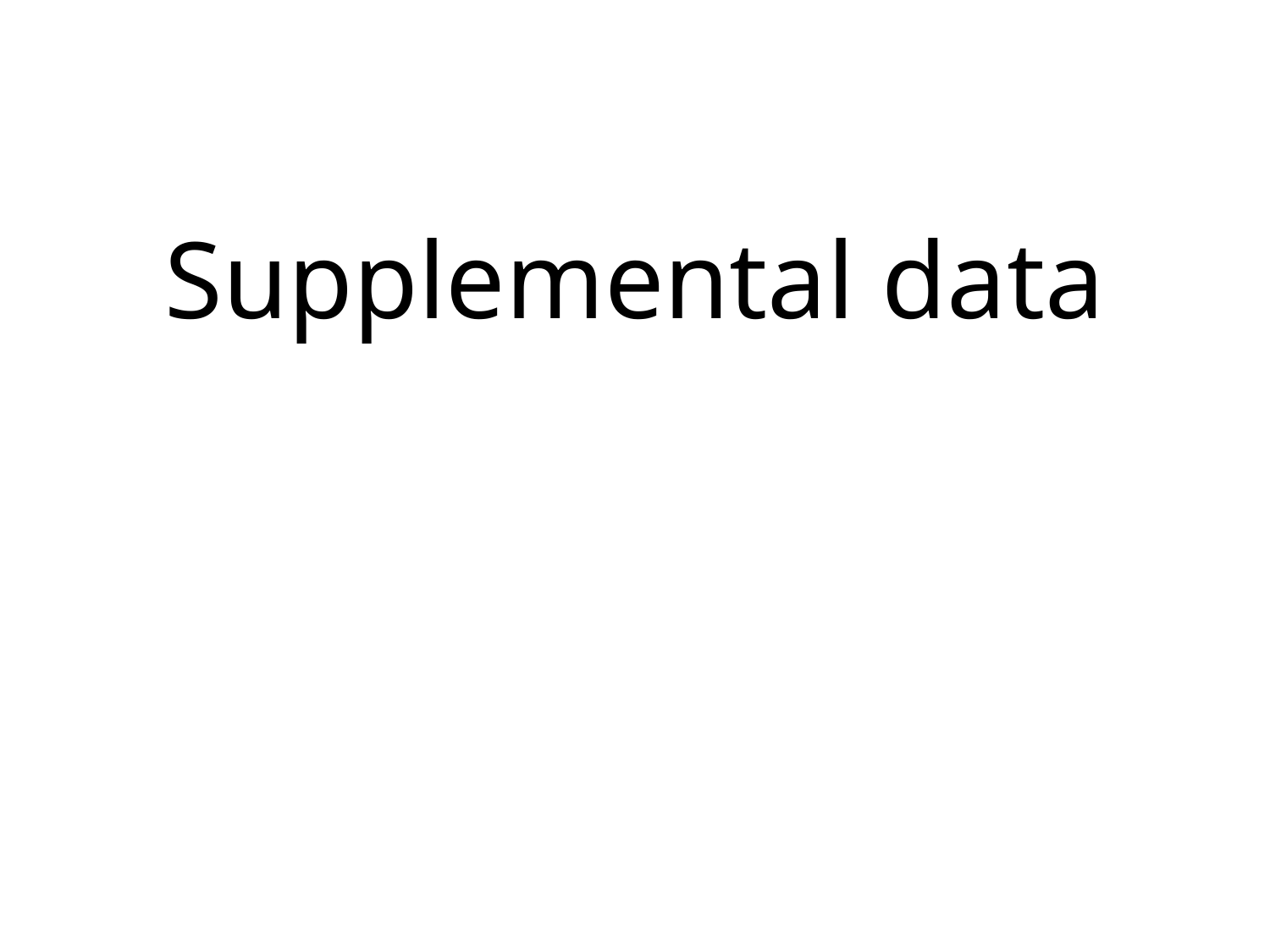

# Supplemental data

## Slide 2
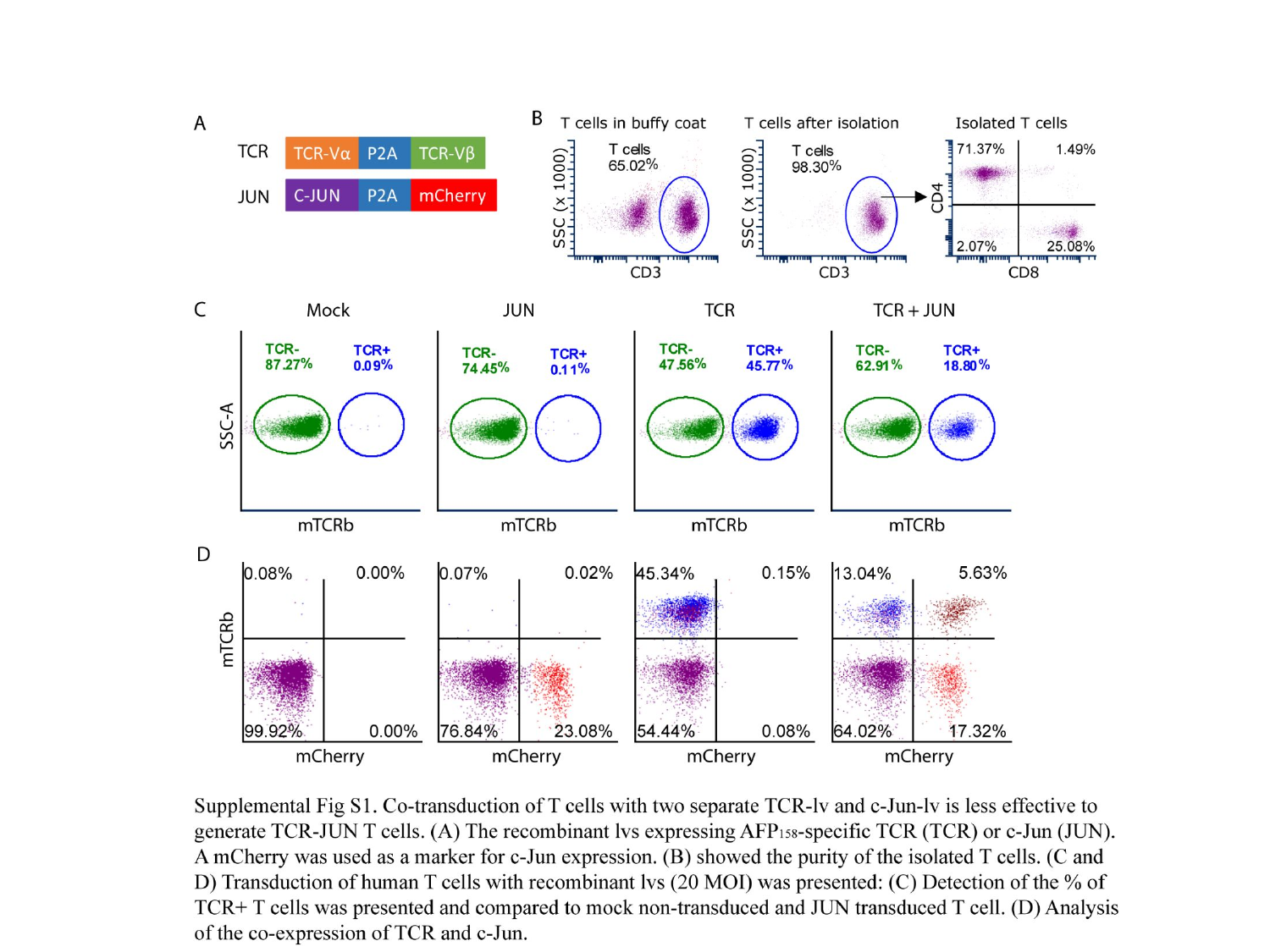

## Slide 3
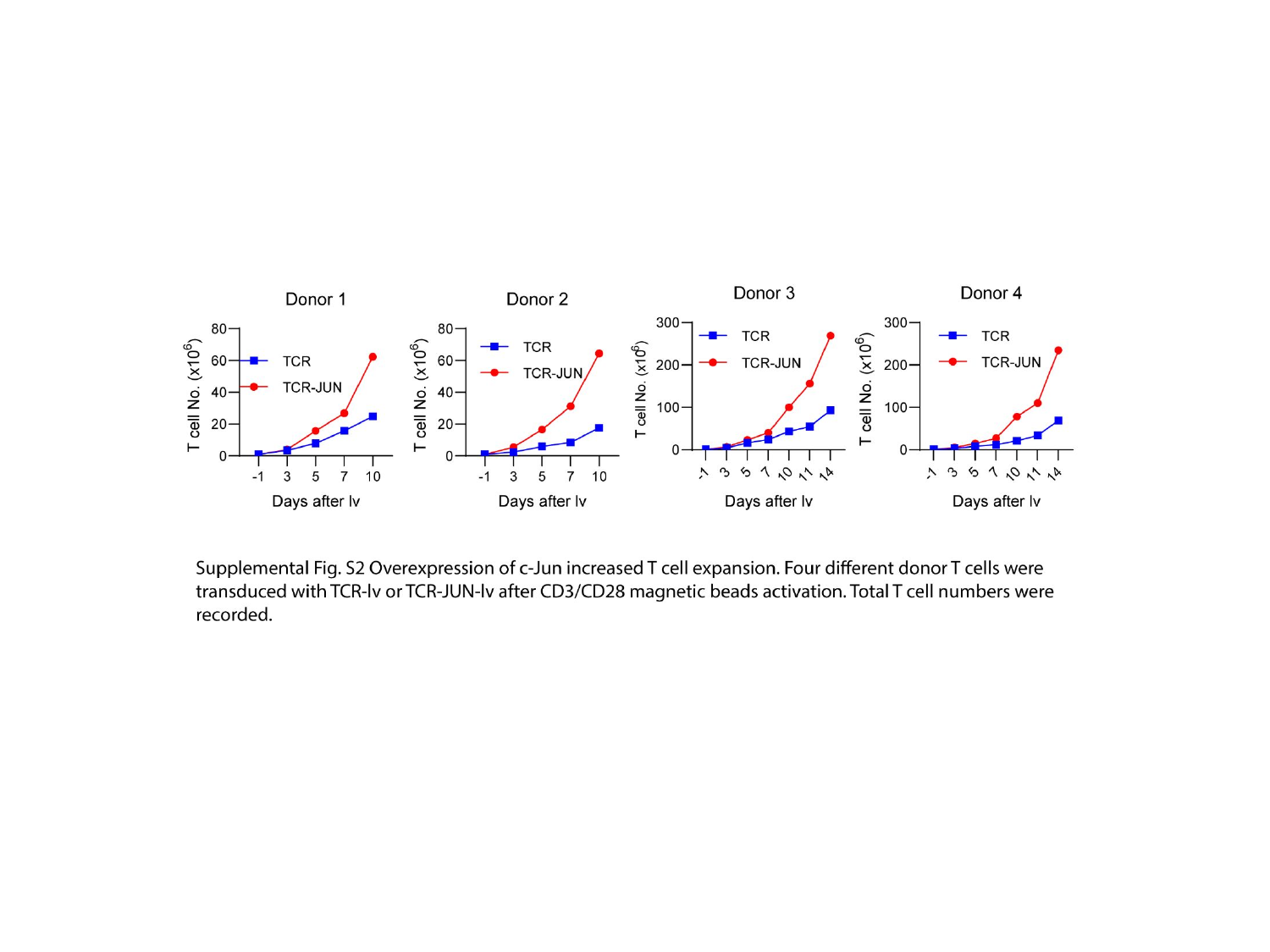

## Slide 4
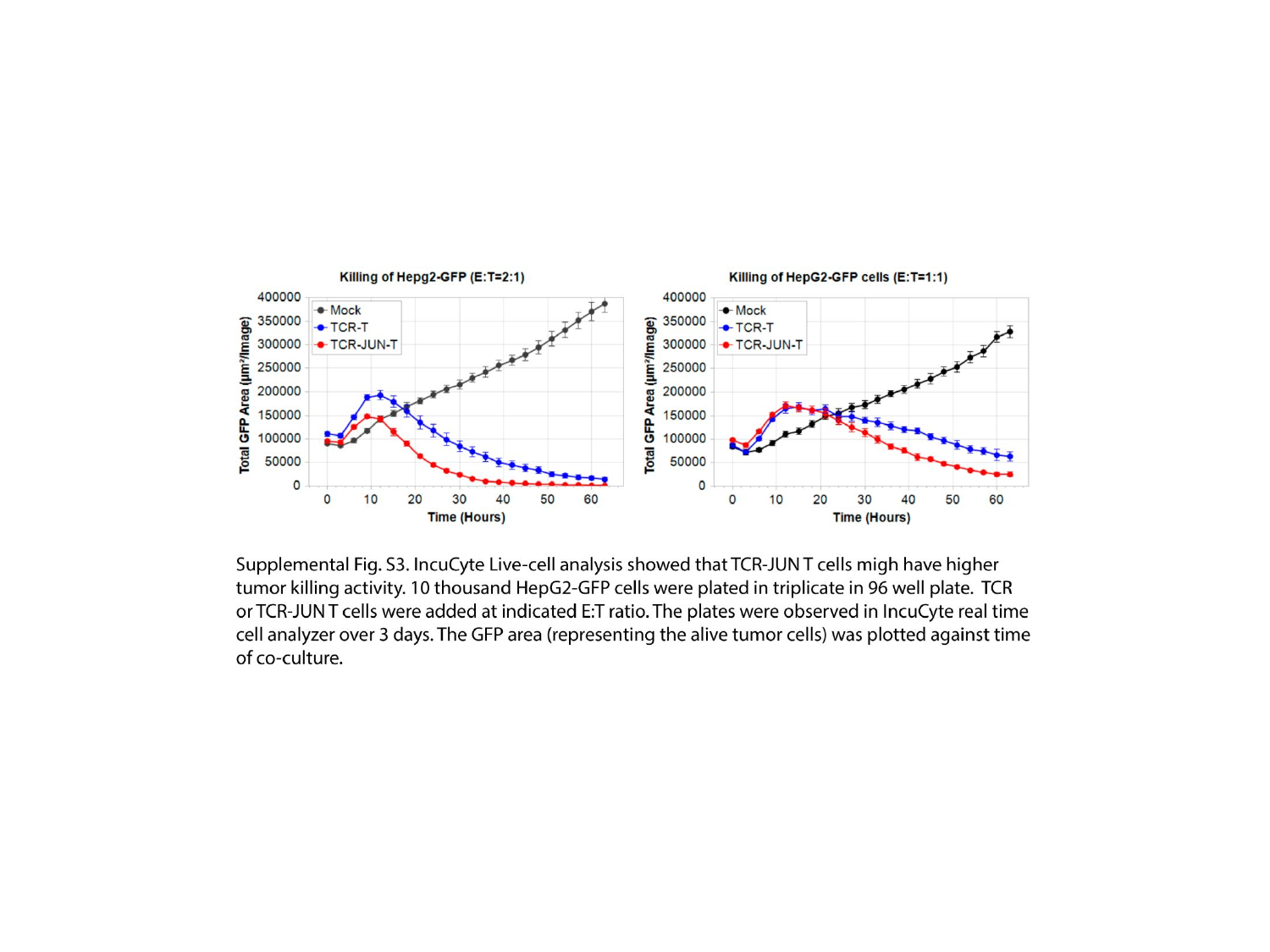

## Slide 5
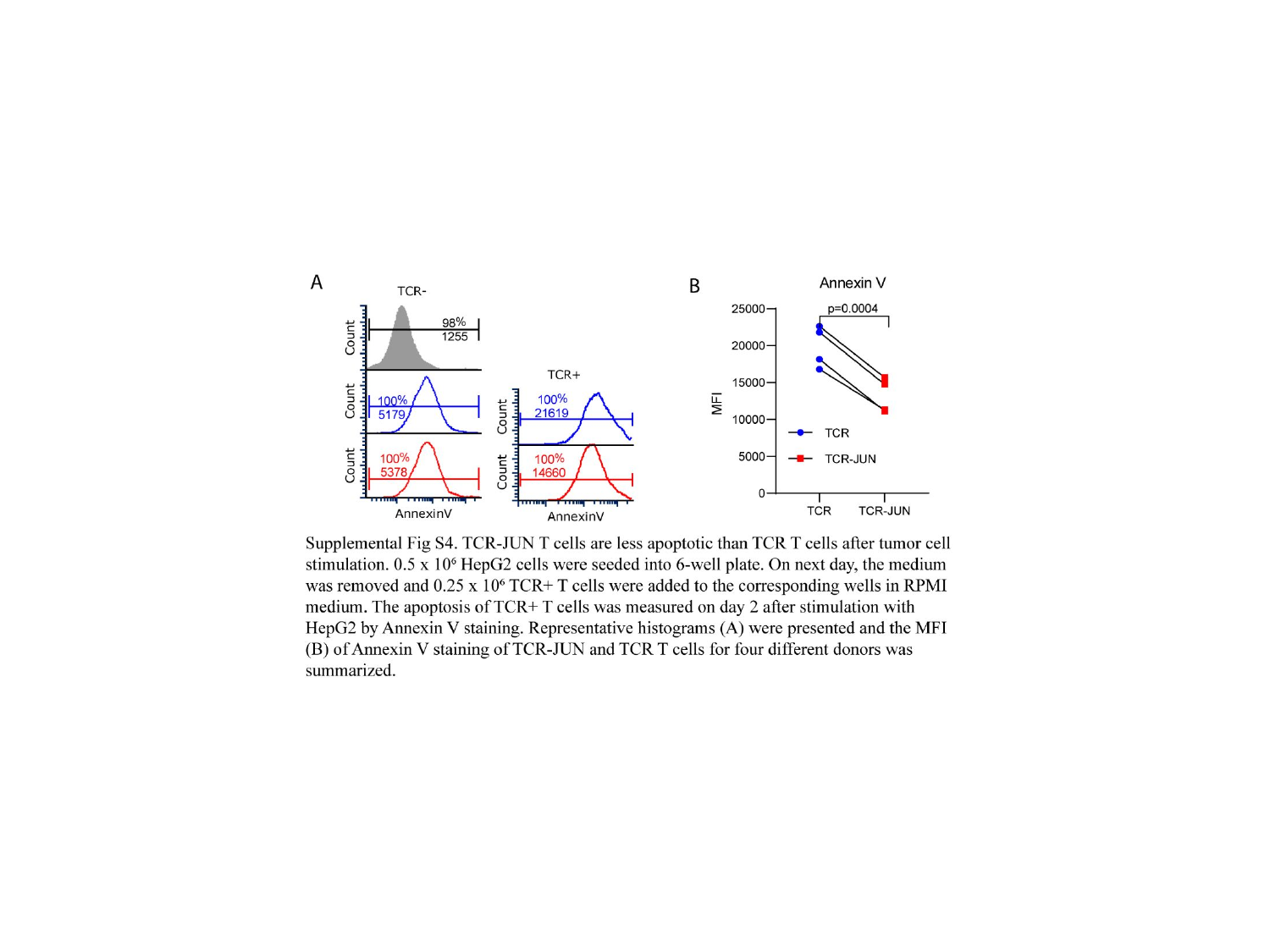

## Slide 6
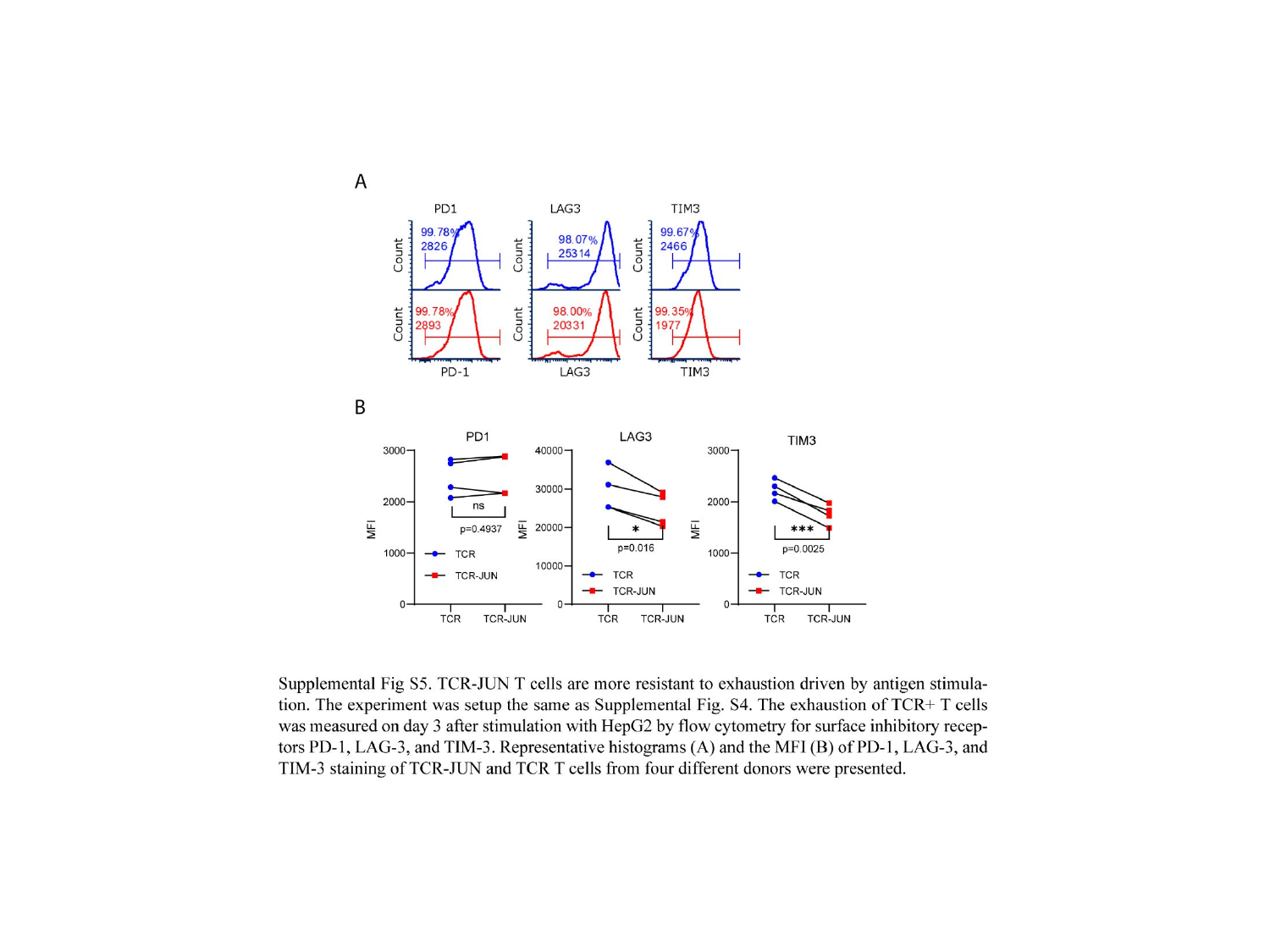

## Slide 7
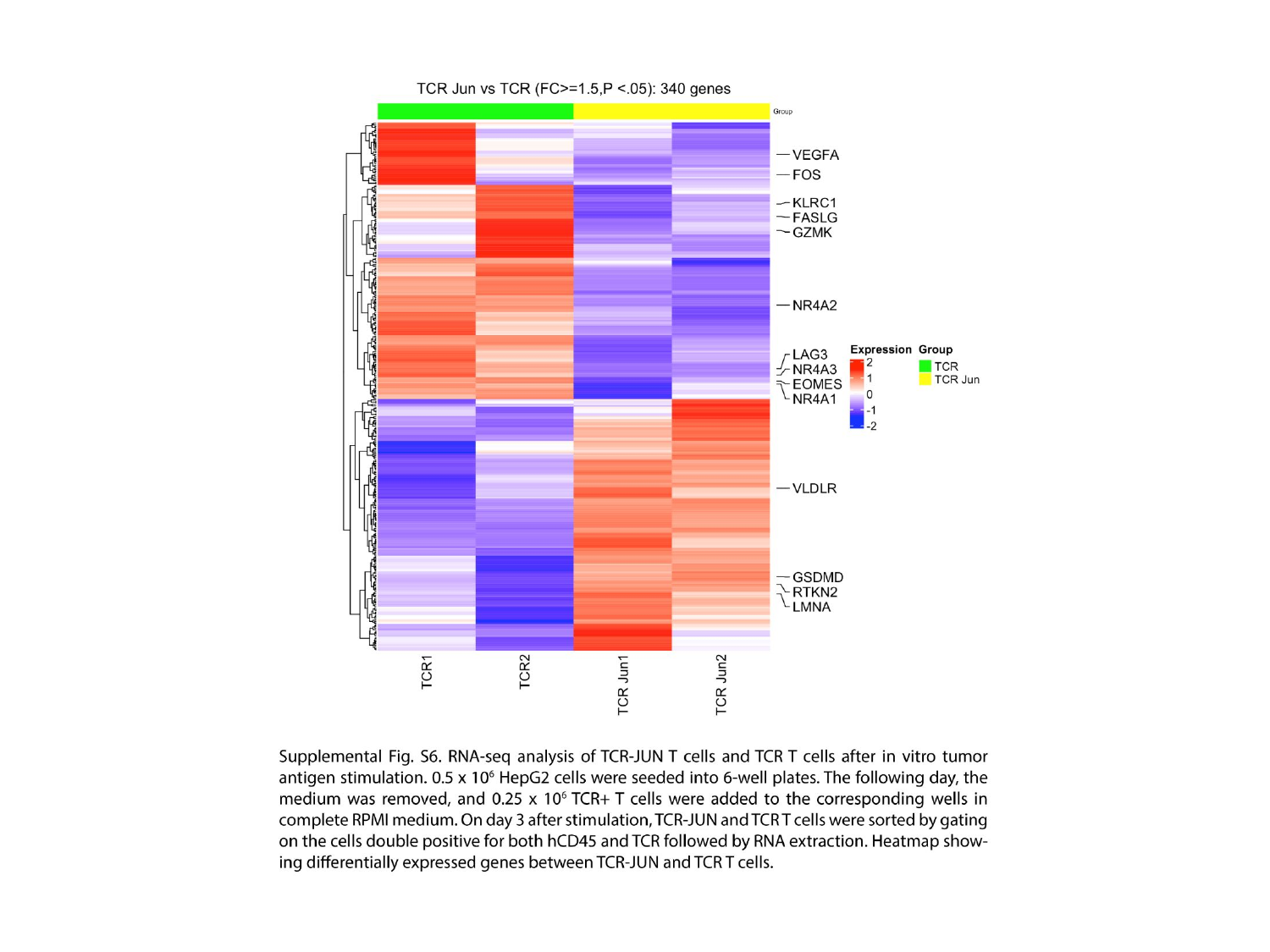

## Slide 8
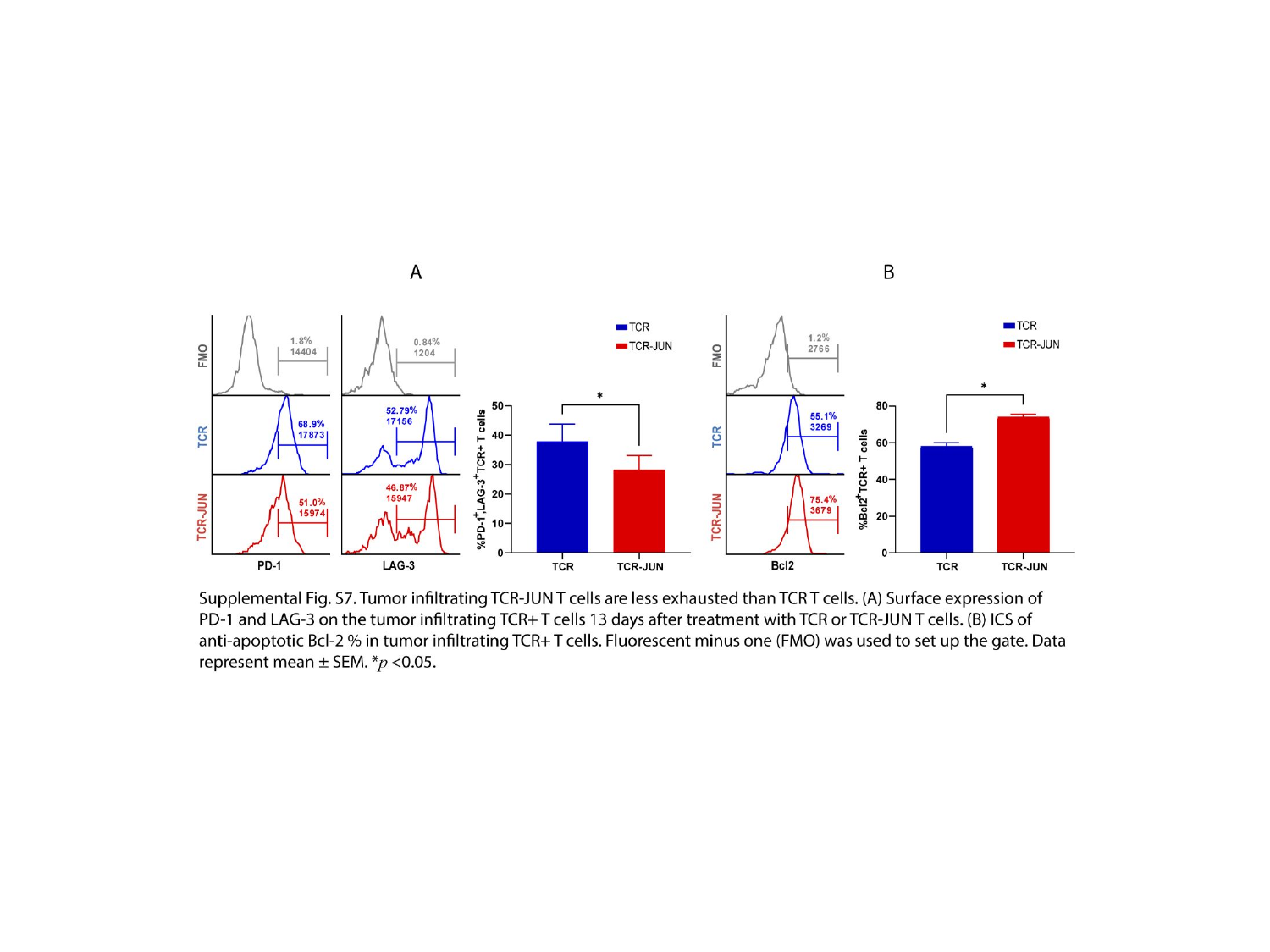

Supplement: Supplementary file 1 [file Presentation_1.pptx]
